# Report of the Royal College of Physicians of London, on Vaccination. With an Appendix, Containing the Opinions of the Royal College of Physcians of Edinburgh and Dublin; and of the Royal Colleges of Surgeons of London, of Dublin, and of Edinburgh

**Published:** 1807-08

**Authors:** Lucas Pepys

**Affiliations:** President. Royal College of Physicians. *Ja. Hervey*, Register


Medical and Physical Journal.

VOL. XVIII.] AUGUST, 1807. [NO. 102.

*Printed for R. PHILLIPS, by W. Thorne, Red Lion Court, Fleet Street, London*

REPORT *of the* ROYAL COLLEGE OF PHYSICIANS *of* LONDON, *on* VACCINATION. *With an* APPENDIX, *containing the Opinions of the Royal College of Physcians of Edinburgh and Dublin; and of the Royal Colleges of Surgeons of London, of Dublin, and of Edinburgh.*

THE Royal College of Physicians of London, having received his Majesty's commands, in compliance with an Address from the House of Commons, "to inquire into the state of Vaccine Inoculation in the United Kingdom, to report their opinion and observations upon that practice, upon the evidence which has been adduced in its support, and upon the Causes which have hitherto retarded its general adoption;" have applied themselves diligently to the business referred to them.

Deeply impressed with the importance of an inquiry which equally involves the lives of individuals and the public prosperity, they have made every exertion to investigate the subject fully and impartially. In aid of the knowledge and experience of the members of their own body, they have applied separately to each of the Licentiates of the College; they have corresponded with the Colleges of Physicians of Dublin and Edinburgh; with the Colleges of Surgeons of London, Edinburgh, and Dublin; they have called upon the Societies established for Vaccination, for an account of their practice, to what extent it has been carried on, and what has been the result of their experience; and they have, by public notice invited individuals to contribute whatever information, they had severally collected. They have in consequence been furnished with a mass of evidence communicated with the greatest readiness and candour, which enables them to speak with confidence upon all the principal points referred to them.

I. During eight years which have elapsed since Dr. Jenner made his discovery public, the progress of Vaccination has been rapid, not only in all parts of the United

Kingdom, but in every quarter of the civilized world. In the British islands some hundred thousands have been vaccinated, in our possessions in the East Indies upwards of 800,000, and among the nations of Europe the practice has become general. Professional men have submitted it to the fairest trials, and the public have, for the most part, received it without prejudice. A few indeed have stood forth the adversaries of Vaccination, on the same grounds as their predecessors who opposed the inoculation for the small-pox, falsely led by hypothetical reasoning in the investigation of a subject which must be supported, or rejected, upon facts and observation only. With these few exceptions, the testimony in favour of vaccination has been most strong and satisfactory, and the practice of it, though it has received a check in some quarters, appears still to be upon the increase in most parts of the United Kingdom.

II. The College of Physicians, in giving their Observations and Opinions on the practice of vaccination, think it right to premise, that they advance nothing but what is supported by the multiplied and unequivocal evidence which has been brought before them, and they have not considered any facts as proved but what have been stated from actual observation.

Vaccination appears to be in general perfectly safe; the instances to the contrary being extremely rare. The disease excited by it is slight, and seldom prevents those under it from following their ordinary occupations. It has been communicated with safety to pregnant women, to children during dentition, and in their earliest infancy; in all which respects it possesses material advantges over inoculation for the small-pox; which, though productive of a disease generally mild, yet sometimes occasions alarming symptoms, and is in a few cases fatal.

The security derived from vaccination against the small-pox, if not absolutely perfect, is as nearly so as can perhaps be expected from any human discovery; for amongst several hundred thousand cases, with the results of which the College have been made acquainted, the number of alleged failures have been surprisingly small, so much so, as to form certainly no reasonable objection to the general adoption of vaccination; for it appears that there is not nearly so many failures in a given number of vaccinated persons, as there are deaths in an equal number of persons inoculated for the small-pox. Nothing can more clearly demonstrate the superiority of vaccination over the inoculation of the small-pox, than this consideration; and it is is a most important fact, which has been confirmed in the course of this inquiry, that in almost every case, where the small-pox has succeeded vaccination, whether by inoculation or by casual infection, the disease has varied much from its ordinary course; it has neither been the same in the violence, nor in the duration of its symptoms, but has, with very few exceptions, been remarkably mild, as if the small-pox had been deprived, by the previous vaccine disease, of all its usual malignity.

The testimonies before the College of Physicians are very decided in declaring, that vaccination does less mischief to the constitution, and less frequently gives rise to other diseases, than the small-pox, either natural or inoculated.

The College feel themselves called upon to state this strongly, because it has been objected to vaccination, that it produces new, unheard-of, and monstrous diseases. Of such assertions no proofs have been produced, and, after diligent inquiry, the College believe them to have been either the inventions of designing, or the mistakes of ignorant men. In these respects then, in its mildness, its safety, and its consequences, the individual may look for the peculiar advantages of vaccination. The benefits which flow from it to society are infinitely more considerable; it spreads no infection, and can be communicated only by inoculation. It is from a consideration of the pernicious effects of the small-pox, that the real value of vaccination is to be estimated. The natural small-pox has been supposed to destroy a sixth part of all whom it attacks; and that even by inoculation, where that has been general in parishes and towns, about one in 300 has usually died. It is not sufficiently known, or not adverted to, that nearly one-tenth, some years more than one-tenth of the whole mortality in London, is occasioned by the small-pox; and however beneficial the inoculation of the small-pox may have been to individuals, it appears to have kept up a constant scource of contagion, which has been the means of increasing the number of deaths by what is called the natural disease. It cannot be doubted that this mischief has been extended by the inconsiderate manner in which great numbers of persons, even since the introduction of vaccination, are still every year inoculated with the small-pox, and afterwards required to attend two or three times a week at the places of inoculation, through every stage of their illness.

From this, then, the public are to expect the great and uncontroverted superiority of vaccination, that it communicates no casual infection, and, while it is a protection to the individual, it is not prejudicial to the public.

III. The College of Physicians, in reporting their observations and opinions on the evidence adduced in support of vaccination, feel themselves authorised to state that a body of evidence so large, so temperate, and so consistent, was perhaps never before collected upon any medical question. A discovery so novel, and to which there was nothing analagous known in nature, though resting on the experimental observations of the inventor, was at first received with diffidence: it was not, however, difficult for others to repeat his experiments, by which the truth of his observations was confirmed, and the doubts of the cautious were gradually dispelled by extensive experience. At the commencement of the practice, almost all that were vaccinated were afterwards submitted to the inoculation of the small-pox; many underwent this operation a second, and even a third time, and the uniform success of these trials quickly bred confidence in the new discovery. But the evidence of the security derived from vaccination against the small-pox does not rest alone upon those who afterwards underwent variolous inoculation, although amounting to many thousands; for it appears, from numerous observations communicated to the College, that those who have been vaccinated are equally secure against the contagion of epidemic small-pox. Towns indeed, and districts of the country, in which vaccination had been general, have afterwards had the small-pox prevalent on all sides of them without suffering from the contagion. There are also in the evidence a few examples of epidemic small-pox having been subdued by a general vaccination. It will not, therefore, appear extraordinary, that many who have communicated their observations should state, that though at first they thought unfavourably of the practice, experience had now removed all their doubts.

It has been already mentioned, that the evidence is not universally favourable, although it is in truth nearly so, for there are a few who entertain sentiments differing widely from those of the great majority of their brethren. The College, therefore, deemed it their duty, in a particular manner to inquire upon what grounds and evidence the opposers of vaccination rested their opinions. From personal examination, as well as from their writings, they endeavoured to learn the full extent and weight of their objections. They found them without experience in vaccination, supporting their opinions by hearsay information, tion, and hypothetical reasoning, and, upon investigating the facts which they advanced, they found them to be either misapprehended or misrepresented; or that they fell under the description of cases of imperfect small-pox, before noticed, and which the College have endeavoured fairly to appreciate.

The practice of vaccination is but of eight years standing, and its promoters, as well as opponents, must keep in mind, that a period so short is too limited to ascertain every point, or to bring the art to that perfection of which it may be capable. The truth of this will readily be admitted by those acquainted with the history of inoculation for the small-pox. Vaccination is now, however, well understood, and its character accurately described. Some deviations from the usual course have occasionally occurred, which the author of the practice has called spurious cow-pox, by which the public have been misled, as if there were a true and a false cow-pox; but it appears, that nothing more was meant, than to express irregularity or difference from that common form and progress of the vaccine pustule from which its efficacy is inferred. Those who perform vaccination ought therefore to be well instructed, and should have watched with the greatest care the regular progress of the pustule, and learnt the most proper time for taking the matter. There is little doubt that some of the failures are to be imputed to the inexperience of the early vaccinators, and it is not unreasonable to expect that farther observation will yet suggest many improvements that will reduce the number of anomalous cases, and furnish the means of determining, with greater precision, when the vaccine disease has been effectually received.

Though the College of Physicians have confined themselves in estimating the evidence to such facts as have occurred in their own country, because the accuracy of them could best be ascertained, they cannot be insensible to the confirmation these receive from the reports of the successful introduction of vaccination, not only into every part of Europe, but throughout the vast Continents of Asia and America.

IV. Several causes have had a partial operation in retarding the general adoption of vaccination; some writers have greatly undervalued the security it affords, while others have considered it to be of a temporary nature only; but if any reliance is to be placed on the statements which have been laid before the College, its power of protecting the human body from the small-pox, though not perfect indeed, is abundantly sufficient to recommend it to the prudent and dispassionate, especially as the small-pox, in the few instances where it has subsequently occurred, has been generally mild and transient. The opinion that vaccination affords but a temporary security is supported by no analogy in nature, nor by the facts which have hitherto occurred. Although the experience of vaccine inoculation be only of a few years, yet the same disease, contracted by the milkers of cows, in some districts has been long enough known to ascertain that in them, at least the unsusceptibility of the small-pox contagion does not wear out by time. Another cause, is the charge against vaccination of producing various new diseases of frightful and monstrous appearance.

Representations of some of these have been exhibited in prints in a way to alarm the feelings of parents, and to infuse dread and apprehension into the minds of the uninformed. Publications with such representations have been widely circulated, and though they originate either in gross ignorance, or wilful misrepresentation, yet have they lessened the confidence of many, particularly of the lower classes, in vaccination; no permanent effects, however, in retarding the progress of vaccination, need be apprehended from such causes, for, as soon as the public shall view them coolly and without surprize, they will excite contempt, and not fear.

Though the College of Physicians are of opinion that the progress of vaccination has been retarded in a few places by the above causes, yet they conceive that its general adoption has been prevented by causes far more powerful, and of a nature wholly different. The lower orders of society can hardly be induced to adopt precautions against evils which may be at a distance; nor can it be expected from them, if these precautions are attended with expence. Unless therefore, from the immediate dread of epidemic small pox, neither vaccination nor inoculation appear at any time to have been general, and when the cause of terror has passed by, the public have relapsed again into a state of indifference and apathy, and the salutary practice has come to a stand. It is not easy to suggest a remedy for an evil so deeply imprinted in human nature. To inform and instruct the public mind may do much, and it will probably be found that the progress of vaccination in different parts of the United Kingdom will be in proportion to that instruction. Were encouragement given to vaccination, by offering it to the poorer poorer classes without expence, there is little doubt but it would in time supersede the inoculation for the small-pox; and thereby various sources of variolous infection would be cut off; but till vaccination becomes general, it will be impossible to prevent the constant recurrence of the natural small-pox by the means of those who are inoculated, except it should appear proper to the legislature to adopt, in its wisdom, some measure by which those who still, from terror or prejudice, prefer the small-pox to the vaccine disease, may, in thus consulting the gratification of their own feelings, be prevented from doing mischief to their neighbours.

From the whole of the above considerations, the College of Physicians feel it their duty strongly to recommend the practice of vaccination. They have been led to this conclusion by no pre-conceived opinion, but by the most unbiassed judgment, formed from an irresistible weight of evidence which has been laid before them. For when the number, the respectability, the disinterestedness, and the extensive experience of its advocates, is compared with the feeble and imperfect testimonies of its few opposers; and when it is considered that many, who were once adverse to vaccination, have been convinced by further trials, and are now to be ranked among its warmest supporters, the truth seems to be established as firmly as the nature of such a question admits; so that the College of Physicians conceive that the public may reasonably look forward with some degree of hope to the time when all opposition shall cease, and the general concurrence of mankind shall at length be able to put an end to the ravages at least, if not to the existence, of the small-pox.

LUCAS PEPYS, President.

Royal College of Physicians
10th April, 1807.

*Ja. Hervey*, Register.

---

### APPENDIX.

### No. 1.

*To the Royal College of Physicians of London.*

GENTLEMEN,

I AM ordered by the King and Queen's College of Physicians, in Ireland, to thank the Royal College of Physicians of London for the communication they have had the honour to receive from them, of certain propositions relative to Vaccination, whereon His Majesty has been pleased to direct an inquiry to be instituted, and in the prosecution of which, the co-operation of the College in Ireland is requested.

And I am directed to acquaint you, that the said College having referred the investigation of these propositions to a Committee, have received from them a Report, of which the enclosed is a copy ; and that they desire the same may be considered as containing their opinion upon the subject

I have the honour to be, Gentlemen,
Your most obedient humble Servant,
HUGH FERGUSON, Register.

By order of the King and Queen's College of Physicians in Ireland.

*Dublin, Nov.* 11, 1806.

" The practice of Vaccination was introduced into this city about the beginning of the year 1801, and appears to have made inconsiderable progress at first. A variety of causes operated to retard its general adoption, amongst which the novelty of the practice, and the extraordinary effects attributed to Vaccination, would naturally take the lead.

" Variolous Inoculation had been long, almost exclusively, in the hands of a particular branch of the profession, whose prejudices and interests were strongly opposed to the new practice ; and by their being the usual medical attendants in families, and especially employed in the diseases of children, their opinions had greater effect upon the minds of parents. The Small-pox is rendered a much less formidable disease in this country by the frequency of Inoculation for it, than it is in other parts of His Majesty's dominions, where prejudices against Inoculation have prevailed ; hence parents, not unnaturally, objected to the introduction of a new disease, rather than not recur to that, with the mildness and safety of which they were well acquainted.

" In the beginning of the year 1804, the Cow-pox Institution was established, under the patronage of the Earl of Hardwicke, and it is from this period that we may date the general introduction of Vaccination into this city, and throughout all parts of Ireland.

" The success of the Institution, in forwarding the new practice, is to be attributed in a great measure to the respectability of the Gentlemen who superintend it, and to the diligence, zeal, and attention of Dr. Labatt, their Secretary and Inoculator. In order to shew the progress which as been made in extending Vaccination, your Committee refer to the Reports of the Cow-pox Institution for the last two years, and to Extracts from their Register for the present year.

| | Patients Inoculated. | Packets issued to Practitioners in general. | Packets to Army Surgeons. |
|---|---|---|---|
| 1804 - | 578 | 776 | 236 |
| 1805 - | 1,032 | 1,124 | 178 |
| 1806 - | 1,356 | 1,340 | 220 |
| Total - | 2,966 | 3,240 | 634 |

" In the above statement, the numbers are averaged to the end of the present year, on the supposition of patients resorting to the Institution as usual. The correspondence of the Institution appears to be very general throughout every part of Ireland, and by the accounts received, as well from Medical Practitioners as others, the success of Vaccination seems to be uniform and effectual. At the present period, in the opinion of your Committee, there are few individuals in any branch of the profession, who oppose the practice of Vaccination in this part of His Majesty's Dominions.

" It is the opinion of your Committee, that the practice of Cow-pox Inoculation is safe, and that it fully answers all the purposes that have been intended by its introduction. At the same time, your Committee is willing to allow that doubtful cases have been reported to them as having occurred, of persons suffering from Small-pox, who had been previously vaccinated. Upon minute investigation, however, it has been found that these supposed instances originated generally in error, misrepresentation, or the difficulty of discriminating between Small-pox and other eruptions, no case having come to the knowledge of your Committee, duly authenticated by respectable and competent judges, of genuine Small-pox succeeding the regular Vaccine disease.

" The practice of Vaccination becomes every day more extended; and, when it is considered that the period at which it came into general use in Ireland is to be reckoned from so late a date, your Committee is of opinion, that it has made already as rapid a progress as could be expected.

(Signed) " James Cleghorn.
" Daniel Mills.
" Hugh Ferguson."

### No. 2.

Physicians Hall, Edinburgh, Nov. 26, 1806.

Gentlemen,

THE Royal College of Physicians of Edinbu gh hav but little opportunity themselves of making observations on

Vaccination, as that practice is entirely conducted by Surgeon Apothecaries, and other Medical Practitioners not of their College, and as the effects produced by it are so inconsiderable and slight, that the aid of a Physician is never required.

The College know that in Edinburgh it is universally approved of by the Profession, and by the higher and middle ranks of the community, and that it has been much more generally adopted by the lower orders of the people than ever the Inoculation for Small-pox was, and they believe the same to obtain all over Scotland.

With regard to any causes which have hitherto prevented its general adoption, they are acquainted with none, except the negligence or ignorance of parents among the common people, or their mistaken ideas of the impropriety or criminality of being accessary to the production of any disease among their children, or the difficulty or impossibility, in some of our Country Districts, of procuring Vaccine matter, or a proper person to inoculate.

The evidence in favour of Vaccination appeared to the Royal College of Physicians of Edinburgh so strong and decisive, that in May last, they spontaneously and unanimously elected Dr. Jenner an Honorary Fellow of their College;—a mark of distinction which they very rarely confer, and which they confine almost exclusively to Foreign Physicians of the first eminence.

They did this with a view to publish their opinion with regard to Vaccination, and in testimony of their conviction of the immense benefits which have been, and which will in future be derived to the world, from Inoculation for the Cow-pox, and as a mark of their sense of Dr. Jenner's very great merits and ability in introducing and promoting this invaluable practice.

I have the honour to be, Gentlemen,
Your most obedient humble Servant,
TH. SPENS, C. R. M. Ed. Pr.

To the Royal College of Physicians of London.

### No. 3.

At a Special Court of Assistants of the Royal College of Surgeons, convened by order of the Master, and holden at the College on Tuesday the 17th day of March 1807;

Mr. Governor LUCAS in the Chair:

Mr. LONG, as Chairman of the Board of Curators, reported, That the Board are now ready to deliver their Report on the subject of vaccination.

It was then moved, seconded, and Resolved, That a Report from the Board of Curators, on the subject of vaccination, which was referred to their consideration by the Court of Assistants, on the 21st day of November last, be now received.

Mr. Long then delivered to Mr. Governor Lucas (presiding in the absence of the Master) a Report from the Board of Curators.

It was then moved, seconded, and Resolved, That the Report delivered by Mr. Long, be now read; and it was read accordingly, and is as follows:

To the Court of Assistants of the Royal College of Surgeons in London.

THE Report of the Board of Curators, on the subject of Vaccination, referred to them by the Court, on the 21st day of November 1806; made to the Court on the 17th of March, 1807.

THE Court of Assistants having received a Letter from the Royal College of Physicians of London, addressed to this College, stating, That His Majesty had been graciously pleased, in compliance with an Address from the Honourable House of Commons, to direct His Royal College of Physicians of London to inquire into the state of Vaccination in the United Kingdom, to Report their Observations and Opinion upon that Practice, upon the Evidence adduced in its support, and upon the Causes which have hitherto retarded its general adoption; that the College were then engaged in the investigation of the several propositions thus referred to them, and requesting this College to co-operate and communicate with them, in order that the Report thereupon might be made as complete as possible.

And having, on the 21st day of November last, referred such letter to the consideration of the Board of Curators, with authority to take such steps respecting the contents thereof as they should judge proper, and report their proceedings thereon, from time to time, to the Court:—The Board proceeded with all possible dispatch to the consideration of the subject.

The Board being of opinion that it would be proper to address circular letters to the Members of this College, with a view of collecting evidence, they submitted to the consideration of the Court, holden on the 15th day of December last, the drafts of such letter as appeared to them best calculated to answer that end; and the same having been approved by the Court, they caused copies thereof to be sent to all the Members of the College in the United Kingdom, whose residence could be ascertained, in the following form; *viz.*

"Sir,

"The Royal College of Surgeons being desirous to co-operate with the Royal College of Physicians of London, in obtaining information respecting vaccination, submit to you the following Questions, to which the favour of your Answer is requested.

"By order of the Court of Assistants.

"Okey Belfour, Secretary."

Lincoln's-Inn Fields, Dec. 15, 1806.

"1st. How many persons have you vaccinated?

"2d. Have any of your patients had the small-pox after vaccination? In the case of every such occurrence, at what period was the vaccine matter taken from the vesicle? How was it preserved? How long before it wa inserted? What was the appearance of the inflammation? And what the interval between vaccination and the variolous eruption?

"3d. Have any bad effects occurred in your experience in consequence of vaccination? And if so, what were they?

"4th. Is the practice of vaccination increasing or decreasing in your neighbourhood; if decreasing, to what cause do you impute it?"

To such letters the Board have received 426 answers; and the following are the results of their investigation:

The number of persons, stated in such letters to have been vaccinated, is 164,381.

The number of cases in which small-pox had followed vaccination is 56.

The Board think it proper to remark under this head, that, in the enumeration of cases in which small-pox has succeeded vaccination, they have included none but those in which the subject was vaccinated by the surgeon reporting the facts.

The bad consequences which have arisen from vaccination are, eruptions of the skin in 66 cases, and inflammation of the arm in 24 instances, of which three proved fatal.

Vaccination, in the greater number of Counties from which Reports have been received, appears to be increasing; it may be proper however to remark, that, in the Metropolis, it is on the decrease.

The principal reasons assigned for decrease are,

Imperfect vaccination,
Instances of small-pox after vaccination,
Supposed bad consequences,
Publications against the practice,
Popular prejudices.

And such Report having een considered, it was moved, seconded, and

Resolved, that the Report now read, be adopted by this Court, as the answer of the Court to the letter of the Royal College of Physicians, of the 23d of October last, on the subject of Vaccination.

Resolved, That a copy of these Minutes and Resolutions, signed by Mr. Governor Lucas (presiding at this Court in the absence of the Master) be transmitted by the Secretary to the Register of the Royal College of Physicians.

(Signed) WM. LUCAS.

### No. 4,

*Edinburgh, March* 3d, 1807.

SIR,

I mentioned in my former letter, that I would take the earliest opportunity of laying before the Royal College of Surgeons of Edinburgh, the communication with which the Royal College of Physicians of London had honoured them, on the 23d of October last:

I am now directed by the Royal College to send the following answer on that important subject.

The practice of vaccine inoculation, both in private and at the Vaccine Institution established here in 1801, is increasing so rapidly, that for two or three years past, the small-pox has been reckoned rather a rare occurrence, even amongst the lower order of the inhabitants of this city, unless in some particular quarters about twelve months ago, and, among the higher ranks of the inhabitants, the disease is unknown.

The Members of the Royal College of Surgeons have much pleasure in reporting, That, as far as their experience goes, they have no doubt of the permanent security against the small-pox which is produced by the constitutional affection of the cow-pox; and that such has hitherto been their success in vaccination, as also to gain for it the confidence of the public, insomuch that they have not been required, for some years past, to inoculate any person with small-pox who have not previously undergone the inoculation with the cow-pox.

The Members of the Royal College have met with no occurrence in their practice of cow-pox inoculation which could operate in their minds to its disadvantage, and they beg leave particularly to notice, that they have seen no instance of obstinate eruptions, or of new and dangerous diseases, which they could attribute to the introduction among mankind of this mild preventive of the small-pox. The Royal College of Surgeons know of no causes which have hitherto retarded the adoption of vaccine inoculation here; on the contrary, the practice has become general within this city: and from many thousand packets of vaccine matter having been sent by the Members of the Royal College, and the Vaccine Institution here, to all parts of the country, the Royal College have reason to believe that the practice has been as generally adopted throughout this part of the United Kingdom as could have been expected from the distance of some parts of the country from proper medical assistance, and other circumstances of that nature.

I have the honour to be, Sir,
Your most obedient servant,
WM. FARQUHARSON,
President of the Royal College and Incorporation of Surgeons of Edinburgh.

### No. 5

Royal College of Surgeons in Ireland,

*Dublin, Feb. 4th,* 1807.

SIR,

I am directed to transmit to you the enclosed Report of a Committee of the College of Surgeons in Ireland, to whom was referred a Letter from the Royal College of Physicians in London, relative to the present state of vaccination in this part of the United Kingdom; and to state, that the College of Surgeons will be highly gratified by more frequent opportunities of corresponding with the English College of Physicians on any subject which may conduce to the advancement of Science, and the welfare of the public.

I have the honour to be, Sir,
Your most obedient humble Servant,
JAMES HENTHORN, Sec.

At a meeting of the Royal College of Surgeons in Ireland, holden at their Theatre, on Tuesday the 13th day of January 1807.

FRANCIS M'EVOY, Esq. President.

Mr. Johnson reported from the committee, to whom was referred a letter from the College of Physicians, London, relative to the present state of Vaccination in the United Kingdom, &c. &c. That they met, and came to the following Resolutions:

That it appears to this Committee, That Inoculation with Vaccine Infection is now very generally adopted by the surgical practitioners in this part of the United Kingdom, as a preventive of Small-pox.

That it appears to this Committee, That from the 25th day of March 1800 to the 25th of November 1806, 11,504 persons have been inoculated with vaccine infection at the dispensary for infant poor, and 2,831 at the cow-pox institution, making a total of 14,335, exclusive of the number inoculated at hospitals and other places, where no registry is made and preserved.

That it is the opinion of this Committee, That the cow-pox has been found to be a mild disease, and rarely attended with danger, or any alarming symptom, and that the few cases of small-pox which have occurred in this country, after supposed vaccination, have been satisfactorily proved to have arisen from accidental circumstances, and cannot be attributed to the want of efficacy in the genuine vaccine infection as a preventive of small-pox.

That it is the opinion of this Committee, That the causes which have hitherto retarded the more general adoption of vaccination in Ireland, have, in a great measure, proceeded from the prejudices of the lower classes of the people, and the interest of some irregular practitioners.

To which Report the College agreed.

Extract from the Minutes.

James Henthorn, Sec.

